# Hospital survival following pediatric HSCT: changes in complications, ICU therapies and outcomes over 10 years

**DOI:** 10.3389/fped.2023.1247792

**Published:** 2023-10-12

**Authors:** Taylor L. Olson, Murray M. Pollack, Blachy J. Dávila Saldaña, Anita K. Patel

**Affiliations:** ^1^ Department of Pediatrics, Division of Critical Care Medicine, Children's National Hospital and George Washington University School of Medicine and Health Sciences, Washington, DC, United States; ^2^ Department of Pediatrics, Division of Blood and Marrow Transplantation, Children's National Hospital and George Washington University School of Medicine and Health Sciences, Washington, DC, United States

**Keywords:** bone marrow transplant, hematopoietic stem cell transplantation, survival, outcomes, intensive care, infection, GVHD

## Abstract

**Introduction:**

Hematopoietic stem cell transplantation (HSCT) is an increasingly utilized therapy for malignant and non-malignant pediatric diseases. HSCT complications, including infection, organ dysfunction, and graft-versus-host-disease (GVHD) often require intensive care unit (ICU) therapies and are associated with mortality. Our aims were to identify the HSCT characteristics, complications and ICU therapies associated with (1) survival, and (2) survival changes over a ten-year period in a national dataset.

**Methods:**

A national sample from the Health Facts (Cerner Corporation, Kansas City, MO) database from 2009 to 2018 was utilized. Inclusion criteria were age 30 days to <22 years and HSCT procedure code. For patients with >1 HSCT, the first was analyzed. Data included demographics, hospital length of stay (LOS), hospital outcome, transplant type and indication. HSCT complications included GVHD and infections. ICU therapies were positive pressure ventilation (PPV), vasoactive infusion, and dialysis. Primary outcome was survival to discharge. Statistical methods included bivariate analyses and multivariate logistic regression.

**Results:**

473 patients underwent HSCT with 93% survival. 62% were allogeneic (89% survival) and 38% were autologous (98% survival). GVHD occurred in 33% of allogeneic HSCT. Infections occurred in 26% of all HSCT. ICU therapies included PPV (11% of patients), vasoactive (25%), and dialysis (3%). Decreased survival was associated with allogeneic HSCT (*p* < 0.01), GVHD (*p* = 0.02), infection (p < 0.01), and ICU therapies (*p* < 0.01). Survival improved from 89% (2009–2013) to 96% (2014–2018) (*p* < 0.01). Allogeneic survival improved (82%–94%, *p* < 0.01) while autologous survival was unchanged. Survival improvement over time was associated with decreasing infections (33%–21%, *p* < 0.01) and increasing vasoactive infusions (20%–28%, *p* = 0.05). On multivariate analysis, later time period was associated with improved survival (*p* < 0.01, adjusted OR 4.28).

**Discussion:**

Hospital survival for HSCT improved from 89% to 96% from 2009 to 2018. Factors associated with mortality included allogeneic HSCT, GVHD, infections and ICU therapies. Improving survival coincided with decreasing infections and increasing vasoactive use.

## Introduction

Hematopoietic stem cell transplantation (HSCT) is an established therapy for children with malignant and non-malignant diseases, including hematologic and solid tumors, bone marrow failure syndromes, immunodeficiencies, and genetic and metabolic disorders (1). As indications for HSCT broadened, transplant volumes have increased by 5%–10% per year; approximately 2,500 children currently undergo HSCT each year in the United States (1–3). However, HSCT carries substantial risk of treatment-related morbidity and mortality, including infectious complications, graft-versus-host disease (GVHD), and organ toxicity induced by preparatory regimens (1, 4, 5).

Approximately one third of patients require intensive care unit (ICU) management for HSCT complications (6–12). Mortality in the first 100 days is as high as 11% for allogeneic transplant and 4% for autologous transplant (1, 3, 5), an improvement from 15% and 7% respectively before 1991 (3). Other studies have revealed similar trends of improving survival over time (5, 13–17). Tracking change is particularly relevant given improvements in human leukocyte antigen-matching, reduced-intensity pre-transplant regimens, GVHD management, infection prophylaxis and treatment (5, 14, 15, 18) and ICU care. Importantly, the contribution of ICU care to these temporal trends has not been evaluated.

Our aims were to associate HSCT characteristics, HSCT complications and ICU therapies with (1) survival, and (2) survival changes over a ten-year time period in a national sample from 2009 to 2018 to assess if survival improved and if there are any changes in HSCT complications or practice associated with improvement.

## Methods

### Database and study design

This is a retrospective multicenter study using the Health Facts™ database (Cerner Corporation, Kansas City, MO). This database has de-identified clinical data from academic and nonacademic hospitals of varied sizes and locations in the United States with a Cerner data use agreement. The database includes demographic and admission information, diagnostic and procedure codes, laboratory results, medication and respiratory data, and hospital outcome. Health Facts™ has been successfully used in other longitudinal studies examining pediatric trends and practice (19, 20). This study was approved by the Children's National Hospital Institutional Review Board (Pro00009282) and granted a waiver of consent for de-identified data.

Inclusion criteria included encounters for patients age 30 days to less than 22 years admitted between January 1, 2009 and June 1, 2018 with at least one HSCT procedure code, indicating receipt of HSCT during the admission. The procedure codes used to define HSCT, associated diagnoses and some therapies are detailed in the Supplementary Appendix A. For patients with more than one HSCT encounter during the study period, only the first was included. Encounters were excluded if they had incomplete data (below).

### Variables and outcome measures

The primary outcome was survival to hospital discharge. Demographic variables included age, sex, race, ethnicity, and hospital length of stay (LOS). HSCT variables included transplant type (autologous and allogeneic), year of transplant, underlying diagnoses necessitating the transplant and complications including GVHD and infection. ICU therapies included positive pressure ventilation (PPV), dialysis, and vasoactive agent infusion. Transplant type, GVHD and underlying diagnosis/indication for HSCT were identified from diagnostic and/or procedure codes. Diagnoses and transplant indications were grouped into categories including malignant hematologic diseases, solid tumors, non-malignant hematologic diseases, immunodeficiencies, and non-malignant other diseases. If more than one diagnosis was present, one was chosen based on clinical expertise and likelihood to necessitate HSCT by T.O and B.D. Infectious complications were identified from microbiology results and were categorized by the culture site as blood, respiratory, urine, skin and soft tissue, or other. Organism types included bacteria (gram positive and gram negative), viruses, and other (fungus, yeast, and mycobacteria); patients could have more than one organism identified. PPV (non-invasive and invasive) was determined from procedure codes and respiratory care data. Respiratory care data indicating PPV included >8 h of recorded ventilator settings. Dialysis (hemodialysis, peritoneal dialysis, urinary filtration, and vascular access for dialysis) was determined from procedure codes. Vasoactive agent infusion (epinephrine, norepinephrine, dopamine, dobutamine, milrinone, and/or vasopressin) was determined from medication administration data.

### Statistical analysis

Variables were assessed individually for their association to hospital survival using bivariate analysis. Bivariate tests included Pearson's *χ*^2^ or Fisher's Exact for categorical variables and Wilcoxon rank sums tests for continuous variables. Post hoc multiple comparisons were performed if the primary comparison was significant.

The study period was divided into two 5-year intervals to assess change over time, 2009–2013 and 2014–2018. Bivariate analyses were performed for demographic, HSCT, ICU care variables, and survival to assess changes between the two time periods.

Multivariable logistic regression was used to investigate the effect of time period, selected demographics, HSCT, and ICU therapy variables on hospital survival. Variables significant at the 0.2 level in the bivariate analyses of survival were included in the multivariable logistic regression model.

Odds ratios and adjusted odds ratios are reported. Statistical significance was declared at the 0.05 alpha level. Results were expressed as medians with 25th–75th percentiles or counts with percentages. All statistical analyses were conducted using JMP® (version 16.1, SAS, Cary, North Carolina, USA).

## Results

**Figure 1:**
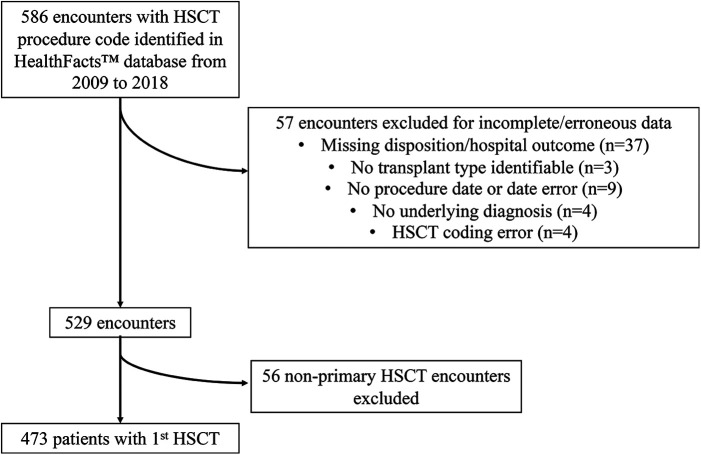
Patient inclusion diagram. HSCT, hematopoietic stem cell transplantation.

A total of 586 encounters met the study inclusion criteria. Fifty-seven encounters were excluded for incomplete data (Figure 1). Only the first HSCT procedure for each patient was included, with 56 subsequent HSCT encounters excluded. The final sample had 473 patients with hospital survival of 93%. Demographic and HSCT variable data are shown in Table 1. Median age was 8 years [3–15]. There were 284 (60%) males, and 322 (68%) patients were Caucasian. Transplant type was allogeneic for 293 patients (62%). Underlying diagnoses and HSCT indications included malignant hematologic diseases (47%), solid tumors (33%), non-malignant hematologic diseases (14%), immunodeficiency syndromes (4%) and other non-malignant diseases (2%). Hospital LOS was 32 days [23–47] and differed between survivors [31 days (23–43)] and deaths [85 days (63–116)] (*p* < 0.01).

**Table 1:** Demographic and hematopoietic stem cell transplant variables and their association with survival.

	All patients (*n* = 473)	Survivors (*n* = 438)	Deaths (*n* = 35)	*p* (1)	OR of Survival [95% CI]
Demographic					
Age, median [25%ile–75%ile], years	8 [3–15]	8 [3–15]	6 [1–15]	0.41 (2)	
Male, *n* (%)	284 (60)	265 (93)	19 (7)	0.47 (3)	1.29 [0.65–2.58]
Female, *n* (%)	189 (40)	173 (92)	16 (8)		
Race and ethnicity, *n* (%)					
Caucasian	322 (68)	299 (93)	23 (7)	0.96 (4)	
African American	69 (15)	63 (91)	6 (9)		
Asian/Pacific Islander	21 (4)	20 (95)	1 (5)		
Hispanic	26 (6)	24 (92)	2 (8)		
Other/Unknown	35 (7)	32 (91)	3 (9)		
Hospital LOS, median [25%ile–75%ile], days	32 [23–47]	31 [23–43]	85 [63–116]	<0.01 (5)	
Transplant Type					
Allogeneic, *n* (%)	293 (62)	262 (89)	31 (11)	<0.01 (6)	0.19 [0.07–0.55]
Autologous, *n* (%)	180 (38)	176 (98)	4 (2)		
Transplant Indication				0.02 (7)	
Malignant Hematologic, *n* (%)	221 (47)	199 (90)	22 (10)	REF (7)	
Solid tumor, *n* (%)	157 (33)	153 (97)	4 (3)	<0.01 (7)	4.23 [1.43–12.53]
Non-malignant Hematologic, *n* (%)	64 (14)	59 (92)	5 (8)	0.81 (7)	1.30 [0.47–3.59]
Immunodeficiency, *n* (%)	21 (4)	18 (86)	3 (14)	0.46 (7)	0.66 [0.18–2.43]
Non-malignant other, *n* (%)	10 (2)	9 (90)	1 (10)	1.00 (7)	0.99 [0.12–8.23]

OR, odds ratio; CI, confidence interval; NS, not significant, LOS, length of stay; REF, reference group.

(1) Continuous variables compared with Wilcoxon rank sums tests. Categorical variables were compared with Pearson's *χ*^2^ or Fisher's Exact, and *post hoc* multiple comparisons were performed when the primary comparison was significant (see Methods).

(2) Comparison of age medians, survivors vs. deaths.

(3) Comparison of sex distributions, survivors vs. deaths.

(4) Comparison of race and ethnicity distributions, survivors vs. deaths.

(5) Comparison of hospital LOS medians, survivors vs. deaths.

(6) Comparison of transplant type distributions, survivors vs. deaths.

(7) Comparison of transplant indication distributions, survivors vs. deaths. Malignant hematologic subgroup served as reference group for *post hoc* multiple comparisons. See Supplementary Appendix for individual diagnoses included in each transplant indication subgroup.

Survival to hospital discharge for allogeneic HSCT was 89% compared to 98% for autologous HSCT [*p* < 0.01, OR 0.19 (0.07–0.55)] (Table 1). Compared to malignant hematologic diseases with 90% survival (reference group), solid tumors had improved survival of 97% [*p* < 0.01, OR 4.23 (1.43–12.53)]. There were no differences in survival between the other HSCT indication groups and the malignant hematologic reference group.

The major complications of GVHD and infections were associated with decreased survival to discharge. GVHD occurred in 96 patients (33% of allogeneic HSCT) and infections in 125 (26% of all HSCT) (Table 2). Survival for allogeneic HSCT patients with GVHD was 83% compared to 92% survival for those without GVHD [*p* = 0.02, OR 0.41 (0.19–0.87)]. Infectious complications were associated with decreased survival [*p* < 0.01, OR 0.27 (0.13–0.54)], with 85% survival if one or more infectious complication occurred and 95% survival if no infectious complications occurred. Among the infectious complication types, positive blood [*p* < 0.01, OR 0.23 (0.11–0.47)], respiratory [*p* < 0.01, OR 0.10 (0.04–0.23)], and urine cultures [*p* = 0.05, OR 0.39 (0.15–1.02)] were associated with decreased survival. The lowest survival (63%) was in patients with a positive respiratory culture.

**Table 2:** Hematopoietic stem cell transplant complications and ICU therapies and their association with survival.

	N (%) (Total *n* = 473)	Survival (%)	*p* (1)	OR of Survival [95% CI]
Complication				
GVHD (2)	96 (33)	80 (83)	0.02	0.41 [0.19–0.87]
No GVHD	197 (67)	182 (92)		
Any infectious complication	125 (26)	106 (85)	<0.01	0.27 [0.13–0.54]
No infectious complication	348 (74)	332 (95)		
Blood culture positive	87 (18)	71 (82)	<0.01	0.23 [0.11–0.47]
No blood culture positive	386 (82)	367 (95)		
Respiratory culture positive	30 (6)	19 (63)	<0.01	0.10 [0.04–0.23]
No respiratory culture positive	443 (94)	419 (95)		
Urine culture positive	39 (8)	33 (85)	0.05	0.39 [0.15–1.02]
No urine culture positive	434 (92)	405 (93)		
Skin/soft tissue culture positive	10 (2)	8 (80)	0.16	0.31 [0.06–1.50]
No skin/soft tissue culture positive	463 (98)	430 (93)		
Other culture positive	11 (2)	9 (82)	0.19	0.35 [0.07–1.67]
No other culture positive	462 (98)	429 (93)		
GVHD plus infectious complication (3)	39 (27)	29 (74)	0.08	0.45 [0.18–1.11]
GVHD or infection alone	105 (73)	91 (87)		
ICU Therapies				
Any ICU Therapy	143 (30)	111 (78)	<0.01	0.03 [0.01–0.11]
No ICU Therapy	330 (70)	327 (99)		
PPV (4)	53 (11)	28 (53)	<0.01	0.03 [0.01–0.06]
No PPV	420 (89)	410 (98)		
Vasoactive infusion (5)	116 (25)	91 (78)	<0.01	0.10 [0.05–0.23]
No vasoactive infusion	357 (75)	347 (97)		
Dialysis (6)	16 (3)	6 (38)	<0.01	0.03 [0.01–0.10]
No Dialysis	457 (97)	432 (95)		
Number of ICU Therapies			<0.01	
1 ICU Therapy (7)	109 (76)	99 (91)	REF	1
2 ICU Therapies	27 (19)	11 (41)	<0.01	0.07 [0.03–0.19]
3 ICU Therapies	7 (5)	1 (14)	<0.01	0.02 [0.002–0.15]

ICU, intensive care unit; OR: odds ratio; CI, confidence interval; GVHD, graft-versus-host-disease; PPV, positive pressure ventilation.

(1) Categorical variables were compared with Pearson's χ^2^ or Fisher's Exact.

(2) Reported as percent of allogeneic transplants (total *n* = 293).

(3) For allogeneic transplant recipients, those with both GVHD and infectious complication were compared to those with GVHD or infectious complication alone (total *n* = 144).

(4) PPV includes invasive and non-invasive modalities.

(5) Vasoactive infusions include epinephrine, norepinephrine, dopamine, dobutamine, milrinone, and/or vasopressin.

(6) Dialysis includes hemodialysis, peritoneal dialysis, urinary filtration and related procedures (see Supplementary Methods and Appendix).

(7) For patients receiving ICU therapies (*n* = 143), receipt of 1, 2, or 3 therapies were compared.

ICU therapies included PPV in 53 patients (11%), vasoactive agent infusion in 116 patients (25%), and dialysis in 16 patients (3%) (Table 2). Survival was 53% for those receiving PPV, 78% for those receiving vasoactive agent infusions, and 38% for those receiving dialysis. Receiving one or more ICU therapies was associated with decreased survival [*p* < 0.01, OR 0.03 (0.01–0.11)]. An increasing number of ICU therapies was associated with worse survival, with 91% survival for one ICU therapy, 41% survival for two ICU therapies, and 14% survival for three ICU therapies (*p* < 0.01).

There was a significant improvement in survival over the 10-year period, from 89% in the early time period (2009–2013) to 96% in the late time period (2014–2018) [*p* < 0.01, OR 2.72 (1.32–5.61)] (Table 3). In particular, allogeneic HSCT survival increased from 82% to 94% [*p* < 0.01, OR 3.51 (1.59–7.77)], while autologous HSCT survival remained unchanged at 98%. The demographic and transplant variables were similar between the time periods with no significant differences in age, sex, transplant type, transplant indication, or LOS. GVHD was not different between the time periods but infectious complications were significantly reduced from 33% to 21% [*p* < 0.01, OR 0.54 (0.36–0.81)]. Of the ICU therapies, there was a trend towards an increase in vasoactive agent infusions from 20% to 28% [*p* = 0.05, OR 1.54 (1.00–2.37)] and a decrease in the use of PPV from 14% to 9% [*p* = 0.07, OR 0.59 (0.33–1.05)].

**Table 3:** Trends over time in hematopoietic stem cell transplant indications, complications, intensive care unit therapies and outcomes.

	Early Time Period (2009–2013) (*n* = 204)	Late Time Period (2014–2018) (*n* = 269)	*p* (1)	Odds Ratio [95% CI]
Demographics				
Age, median [25%ile–75%ile], years	7 [3–15]	8 [3–14]	0.73	
Male (*n*, column %)	116 (57)	168 (62)	0.22	0.79 [0.55–1.15]
Female (*n*, column %)	88 (43)	101 (38)		
Transplant type				
Allogeneic (*n*, column %)	119 (58)	174 (65)	0.16 (2)	0.76 [0.53–1.11]
Autologous (*n*, column %)	85 (42)	95 (35)		
Hospital outcome				
Overall				
Survivors (*n*, column %)	181 (89)	257 (96)	<0.01 (3)	2.72 [1.32–5.61]
Deaths (*n*, column %)	23 (11)	12 (4)		
Allogeneic				
Survivors (*n*, %allogeneic/column)	98 (82)	164 (94)	<0.01 (4)	3.51 [1.59–7.77]
Deaths (*n*, %allogeneic/column)	21 (18)	10 (6)		
Autologous				
Survivors (*n*, % autologous/column)	83 (98)	93 (98)	1.00 (5)	1.12 [0.15–8.13]
Deaths (*n*, % autologous/column)	2 (2)	2 (2)		
Hospital LOS, median [25%ile–75%ile], days	31 [23–47]	33 [24–48]	0.47	
Transplant Indication (6)			0.10	
Malignant Hematologic (*n*, column %)	83 (41)	138 (51)		
Solid tumor (*n*, column %)	76 (37)	81 (30)		
Non-malignant Hematologic (*n*, column %)	29 (14)	35 (13)		
Immunodeficiency (*n*, column %)	9 (4)	12 (4)		
Non-malignant other (*n*, column %)	7 (3)	3 (1)		
Transplant Complication				
GVHD (*n*, %allogeneic transplant/column) (7)	39 (33)	57 (33)	1.00	1.00 [0.61–1.64]
No GVHD (*n*, %allogeneic transplant/column) (7)	80 (67)	117 (67)		
Any infectious complication (*n*, column %)	68 (33)	57 (21)	<0.01	0.54 [0.36–0.81]
No Infectious complication (*n*, column %)	136 (67)	212 (79)		
ICU Therapies				
Any ICU Therapy (*n*, column %)	58 (28)	85 (32)	0.46	0.16 [0.78–1.73]
No ICU Therapy (*n*, column %)	146 (72)	184 (68)		
PPV (*n*, column %)	29 (14)	24 (9)	0.07	0.59 [0.33–1.05]
No PPV (*n*, column %)	175 (86)	245 (91)		
Dialysis (*n*, column %)	8 (4)	8 (3)	0.57	0.75 [0.28–2.04]
No Dialysis (*n*, column %)	196 (96)	261 (97)		
Vasoactive infusion (*n*, column %)	41 (20)	75 (28)	0.05	1.54 [1.00–2.37]
No Vasoactive infusion (*n*, column %)	163 (80)	194 (72)		

CI, confidence interval; NS, not significant; LOS, length of stay; GVHD, graft-versus-host-disease; ICU, intensive care unit; PPV, positive pressure ventilation.

(1) Variable distributions in the early versus late time periods were compared. Continuous variables were compared with Wilcoxon rank sums tests, and categorical variables were compared with Pearson's χ^2^ or Fisher's Exact.

(2) Comparison of transplant type distribution (allogeneic/autologous), early vs. late time periods.

(3) Comparison of survival distribution for all transplant types, early vs. late time periods.

(4) Comparison of survival distribution for allogeneic transplants, early vs. late time periods.

(5) Comparison of survival distribution for autologous transplants, early vs. late time periods.

(6) See Supplementary Appendix for individual diagnoses included in each transplant indication subgroup.

(7) GVHD reported as percent of allogeneic transplants (total *n* = 293).

The results of the multivariable logistic regression investigating the effect of time period on hospital survival, controlled for HSCT type, indication, GVHD, infectious complications and ICU therapies, are shown in Table 4 (Online). The adjusted OR for survival in the late time period relative to the early time period was 4.44 [1.43–13.77] (*p* < 0.01). ICU therapies were associated with decreased survival on multivariate analysis including PPV [*p* < 0.01, adjusted OR 0.07 (0.02–0.19)], vasoactive infusion [*p* < 0.01, adjusted OR 0.08 (0.03–0.24)], and dialysis [*p* = 0.01, adjusted OR 0.12 (0.02–0.68)].

**Table 4:** Hematopoietic stem cell transplant variables including transplant time period and their association with survival: A multivariable logistic regression.

Variable	*p*	aOR Survival (95% CI)
PPV	<0.01	0.07 [0.02–0.19]
Vasoactive infusion	<0.01	0.08 [0.03–0.24]
Time period (2014–2018)	<0.01	4.44 [1.43–13.77]
Dialysis	0.01	0.12 [0.02–0.68]
Transplant type/GVHD		
Autologous transplant	REF	1
Allogeneic transplant without GVHD	0.61	0.41 [0.01–12.71]
Allogeneic transplant with GVHD	0.36	0.19 [0.01–6.51]
Infectious Complication	0.56	0.72 [0.24–2.15]
Transplant Indication		
Malignant Hematologic	REF	1
Solid tumor	0.84	0.70 [0.02–22.21]
Non-malignant Hematologic	0.50	0.60 [0.14–2.61]
Immunodeficiency	0.61	1.60 [0.26–10.00]
Non-malignant other	0.43	2.99 [0.20–45.37]

aOR, adjusted odds ratio; CI, confidence interval; PPV, positive pressure ventilation; GVHD, graft-versus-host-disease; REF, reference group.

## Discussion

We observed a 93% survival after hospital admission for pediatric HSCT in a large multicenter sample in the United States from 2009 to 2018. Survival was 89% for allogeneic HSCT and 98% for autologous HSCT. Clinical variables associated with decreased survival included allogeneic HSCT, complications of GVHD and infection, and indicators of severity of illness post-HSCT including ICU therapies of PPV, vasoactive agent infusion and dialysis. Survival significantly improved from 89% (2009–2013) to 96% (2014–2018); in particular allogeneic HSCT survival improved (82%–94%) while autologous HSCT survival remained unchanged. Survival improvement was accompanied by decreasing infectious complications and increasing vasoactive agent use over time. After adjusting for HSCT variables, HSCT complications, and ICU therapies in a multivariable regression, time period was a significant predictor of survival (*p* < 0.01) with an adjusted OR of 4.44 [1.43–13.77].

Early treatment-related mortality, often standardized to 100 days following HSCT, is generally attributable to organ toxicity from the transplant conditioning regimen, infection during the period of immunosuppression, and GVHD, as opposed to relapse-related mortality which generally occurs later post-transplant. Since there is no risk of GVHD for autologous HSCT, there is no need for prophylactic immunosuppression with decreased risk of infection as a result (21). Mortality at 100 days is as high as 11% for allogenic HSCT and 4% for autologous HSCT (1, 3, 5) which has improved over time (3, 5, 13–16). We observed 11% and 2% hospital mortality for allogeneic and autologous HSCT respectively at median hospital day 85 [63–116]. Because these data were acquired from a multi-institutional database, we used HSCT admission hospital survival as a proxy for early (100-day) treatment-related mortality.

These findings support the trends of decreasing HSCT complications and improving survival noted over the last several decades (3–5, 13, 14, 16, 17, 22–25). Outcome improvement over time is presumably related, in part, to advancements in HSCT care including reduced intensity conditioning (3, 5, 15, 24, 26) higher resolution human leukocyte antigen-matching (18, 27, 28), expanded agents for bacterial, viral and fungal prophylaxis and treatment, enhanced detection of infection (29–36), and novel GVHD prophylaxis and treatment strategies (14, 24, 37–42). In particular, we identified that infectious complications were significantly reduced over time which was temporally associated with improving survival over time. However, infectious complications were still frequent and were associated with decreased survival, with the worst survival seen for respiratory infections (63%). Respiratory infections have a high mortality in HSCT patients (43, 44) and both animal and human data suggest defects in the pulmonary immune response following HSCT may be contributing (45).

A total of 17%–35% of children require ICU care following HSCT (9–12) and outcomes for post-HSCT ICU patients have improved over time in parallel with HSCT survival (46, 47). For instance, survival of mechanically ventilated HSCT patients has steadily increased from 9%–14% in the 1970–1980s (48–50), to 12%–47% in the 1980–1990s (51–54), and to 18%–58% in the 1990s to early 2000s (6, 10, 17, 47, 55, 56) with current estimates of 39%–58% survival (8, 9, 11, 57–60). In our 2009–2018 cohort, 30% of patients received at least one ICU therapy in the immediate post-HSCT period. Survival was 53% for patients receiving PPV, 78% for patients receiving at least one vasoactive agent infusion, and 38% for patients receiving dialysis, comparable to other recent studies (8, 9, 57, 58). We also found that survival decreased with an increasing number of ICU therapies received: 91% of patients receiving only one therapy survived, while 41% of those receiving two therapies survived, and 14% of those receiving all three therapies survived.

Our observation that increased vasoactive agent use was temporally associated with improvement in survival is novel and may represent a practice shift towards more liberal vasoactive use. Emphasis on early recognition of sepsis with prompt initiation of vasoactive treatments, including peripheral delivery (a modification to guidelines in 2007) (61), may have contributed to increasing use and be partly responsible for this observation. This finding may also relate to the potential harmful effects of fluid overload post-HSCT and recommendations for conservative fluid management (62–66) which could have influenced increasing use of vasoactive agent infusions. Additionally, we observed a trend towards decreasing use of PPV over time during the study period. Decreasing use of mechanical ventilation in this population has been previously reported (17, 67) in conjunction with improving survival. Decreasing infections (that may manifest as deterioration requiring PPV) may be contributing. The impact of non-invasive PPV use on this trend is unclear. In our cohort, only 3 patients received only non-invasive PPV, limiting our inferences on the overall PPV trend.

There are limitations to this study. First, national databases, while providing large samples from multiple sites, usually lack the granularity present in single site data. Therefore, some important HSCT variables could not be analyzed, including donor source, matching, and conditioning regimen. Second, our use of positive culture results as evidence of infectious complications did not include clinical corroboration and presumably missed culture-negative infections or mis-assigned instances of contamination. Third, respiratory cultures may be more likely sent for mechanically ventilated patients, contributing to the low survival seen in this group. Fourth, while we were able to assess ICU therapies (PPV, vasoactive agent infusion, dialysis), we were not able to assess other details of ICU care such as admission and discharge dates, indications for admission or therapies, or other measures of severity of illness. Comorbid diagnoses and some therapies were deduced from diagnosis and procedure codes, potentially missing those that were not coded/billed. For example, the specific diagnostic code for hepatic veno-occlusive disease was introduced in 2015 and therefore was not assessed in this study. Finally, we could not ascertain cause of death or outcome after discharge (including 100-day mortality for survivors discharged before 100 days).

## Conclusion

Hospital survival following HSCT was 93% in a recent multicenter national sample from 2009 to 2018. Factors associated with decreased survival included allogeneic HSCT, GVHD, infectious complications and ICU therapies. Survival significantly improved over time, from 89% to 96%, particularly for allogeneic HSCT. In addition, improving survival was associated with decreasing infectious complications and increasing vasoactive agent use.

## Data Availability

The raw data supporting the conclusions of this article will be made available by the authors, without undue reservation.
