# Supplementary material for: Hospital survival following pediatric HSCT: changes in complications, ICU therapies and outcomes over 10 years
Source: Front Pediatr. 2023 Oct 12;11:1247792. doi: 10.3389/fped.2023.1247792 (PMC10601648; doi:10.3389/fped.2023.1247792)
Supplement: Supplementary file 1 [file Datasheet1.pdf]

Supplementary Material

Hospital survival following pediatric HSCT: changes in complications, ICU therapies and outcomes over 10 years

Taylor L. Olson MD*, Murray M. Pollack MD, Blachy J. Dávila Saldaña MD, Anita K. Patel MD

***Correspondence:** Taylor Olson: tolson@childrensnational.org

Appendix A: Classification of diagnostic and procedure codes appearing in the dataset

Allogeneic Hematopoietic Stem Cell Transplantation:
<u>ICD-9-CM:</u> 41; 41.02; 41.03; 41.05; 41.06
<u>CPT 4:</u> 38240; 38242
<u>ICD-10-PCS:</u> 30233G1; 30233G3; 30233X3; 30233Y2; 30233Y3; 30233Y4; 30240G2; 30240G3; 30243G1; 30243G2; 30243G3; 30243X1; 30243X3; 30243Y1; 30243Y2; 30243Y3; 30243Y4; 30253Y1; 30263G1; 30263X1

Autologous Hematopoietic Stem Cell Transplantation:
<u>ICD-9-CM:</u> 41.01; 41.04; 41.07
<u>CPT 4:</u> 38241
<u>ICD-10-PCS:</u> 30230G0; 30233G0; 30233Y0; 30243G0; 30243Y0

Graft-versus-host-disease:
<u>ICD 9:</u>
279.51 (Acute graft-versus-host disease);
279.52 (Chronic graft-versus-host disease);
279.53 (Acute on chronic graft-versus-host disease);
279.5 (Graft-versus-host disease, unspecified)

<u>ICD-10:</u>
D89.810 (Acute graft-versus-host disease);
D89.811 (Chronic graft-versus-host disease);
D89.812 (Acute on chronic graft-versus-host disease);
D89.813 (Graft-versus-host disease, unspecified)

Positive Pressure Ventilation:
<u>ICD 9-CM:</u>
93.9 (Non-invasive mechanical ventilation);
96.04 (Insertion of endotracheal tube);
96.71 (Continuous invasive mechanical ventilation for less than 96 consecutive hours);
96.72 (Continuous invasive mechanical ventilation for 96 consecutive hours or more)

<u>CPT4:</u>
31500 (Intubation, endotracheal, emergency procedure)

ICD-10-PCS:
0BH17EZ (Insertion of Endotracheal Airway into Trachea, Via Natural or Artificial Opening);
5A1945Z (Respiratory Ventilation, 24-96 Consecutive Hours);
5A1955Z (Respiratory Ventilation, Greater than 96 Consecutive Hours);

Dialysis:
ICD 9-CM:
38.95 (Venous catheterization for renal dialysis);
39.95 (Hemodialysis);
54.98 (Peritoneal dialysis)

CPT4: 90935 (Hemodialysis procedure with single physician evaluation)

ICD-10-PCS: 5A1D60Z (Performance of Urinary Filtration, Multiple)

Malignant Hematologic Disease:

Leukemia
Acute Lymphoid Leukemia
ICD9:
204 Acute lymphoid leukemia without mention of having achieved remission;
204.01 Acute lymphoid leukemia in remission;
204.02 Acute lymphoid leukemia in relapse

ICD10:
C91.00 Acute lymphoblastic leukemia not having achieved remission;
C91.01 Acute lymphoblastic leukemia, in remission;
C91.02 Acute lymphoblastic leukemia, in relapse

Acute myeloid leukemia
ICD9:
205 Acute myeloid leukemia without mention of having achieved remission;
205.01 Acute myeloid leukemia in remission;
205.02 Acute myeloid leukemia in relapse;
205.3 Myeloid sarcoma without mention of having achieved remission;
206 Acute monocytic leukemia without mention of having achieved remission;
207.2 Megakaryocytic leukemia without mention of having achieved remission

ICD10:
C92.00 Acute myeloblastic leukemia, not having achieved remission;
C92.01 Acute myeloblastic leukemia, in remission;
C92.02 Acute myeloblastic leukemia, in relapse;
C92.40 Acute promyelocytic leukemia, not having achieved remission;
C92.50 Acute myelomonocytic leukemia, not having achieved remission;
C93.00 Acute monoblastic/monocytic leukemia, not having achieved remission;

C93.90 Monocytic leukemia, unspecified, not having achieved remission;
C94.20 Acute megakaryoblastic leukemia not having achieved remission;
C94.21 Acute megakaryoblastic leukemia, in remission;

Other leukemia
ICD9:
204.8 Other lymphoid leukemia without mention of having achieved remission;
204.9 Unspecified lymphoid leukemia without mention of having achieved remission;
205.1 Chronic myeloid leukemia without mention of having achieved remission;
205.11 Chronic myeloid leukemia in remission;
205.12 Chronic myeloid leukemia in relapse;
205.8 Other myeloid leukemia without mention of having achieved remission;
205.82 Other myeloid leukemia in relapse;
205.9 Unspecified myeloid leukemia without mention of having achieved remission;
205.91 UNSPECIFIED MYELOID LEUKEMIA IN REMISSION;
205.92 Unspecified myeloid leukemia in relapse;
206.8 Other monocytic leukemia without mention of having achieved remission;
207.81 Other specified leukemia in remission;
207.82 Other specified leukemia in relapse;
208 Acute leukemia of unspecified cell type without mention of having achieved remission;
208.8 Other leukemia of unspecified cell type without mention of having achieved remission;
208.9 Unspecified leukemia without mention of having achieved remission;

ICD10:
C92.10 Chronic myeloid leukemia, BCR/ABL-positive, not having achieved remission
C92.11 Chronic myeloid leukemia, BCR/ABL-positive, in remission
C92.12 Chronic myeloid leukemia, BCR/ABL-positive, in relapse
C92.90 Myeloid leukemia, unspecified, not having achieved remission
C92.91 Myeloid leukemia, unspecified in remission
C92.92 Myeloid leukemia, unspecified in relapse
C92.Z0 Other myeloid leukemia not having achieved remission
C92.Z2 Other myeloid leukemia, in relapse
C94.81 Other specified leukemias, in remission
C95.00 Acute leukemia of unspecified cell type not having achieved remission
C95.11 Chronic leukemia of unspecified cell type, in remission
C95.90 Leukemia, unspecified not having achieved remission

Lymphoma
Hodgkin's lymphoma
ICD9:
201.18 Hodgkin's granuloma involving lymph nodes of multiple sites;
201.13 Hodgkin's granuloma involving intra-abdominal lymph nodes;
201.5 Hodgkin's disease, nodular sclerosis, unspecified site, extranodal and solid organ sites
201.51 Hodgkin's disease, nodular sclerosis, involving lymph nodes of head, face, and neck;
201.52 Hodgkin's disease, nodular sclerosis, involving intrathoracic lymph nodes;
201.53 Hodgkin's disease, nodular sclerosis, involving intra-abdominal lymph nodes;

201.58 Hodgkin's disease, nodular sclerosis, involving lymph nodes of multiple sites;
201.9 Hodgkin's disease, unspecified type, unspecified site, extranodal and solid organ sites
201.92 Hodgkin's disease, unspecified type, involving intrathoracic lymph nodes;
201.93 Hodgkin's disease, unspecified type, involving intra-abdominal lymph nodes;
201.98 Hodgkin's disease, unspecified type, involving lymph nodes of multiple sites

ICD10:
C81.11 Nodular sclerosis Hodgkin lymphoma, lymph nodes of head, face, and neck;
C81.12 Nodular sclerosis Hodgkin lymphoma, intrathoracic lymph nodes;
C81.90 Hodgkin lymphoma, unspecified, unspecified site;
C81.91 Hodgkin lymphoma, unspecified, lymph nodes of head, face, and neck;
C81.98 Hodgkin lymphoma, unspecified, lymph nodes of multiple sites

Non-Hodgkin's lymphoma
ICD9:
200.1 Lymphosarcoma, unspecified site, extranodal and solid organ sites;
200.13 Lymphosarcoma involving intra-abdominal lymph nodes;
200.2 Burkitt's tumor or lymphoma, unspecified site, extranodal and solid organ sites;
200.21 Burkitt's tumor or lymphoma involving lymph nodes of head, face, and neck;
200.23 Burkitt's tumor or lymphoma involving intra-abdominal lymph nodes;
200.38 Marginal zone lymphoma involving lymph nodes of multiple sites;
200.5 Primary central nervous system lymphoma, unspecified site, extranodal and solid organ sites;
200.6 Anaplastic large cell lymphoma, unspecified site, extranodal and solid organ sites;
200.61 Anaplastic large cell lymphoma involving lymph nodes of head, face, and neck;
200.68 Anaplastic large cell lymphoma involving lymph nodes of multiple sites;
200.7 Large cell lymphoma, unspecified site, extranodal and solid organ sites;
200.77 Large cell lymphoma involving spleen;
200.78 Large cell lymphoma involving lymph nodes of multiple sites;
200.8 Other named variants of lymphosarcoma and reticulosarcoma and other specified malignant tumors of lymphatic tissue, unspecified site, extranodal and solid organ sites;
202.1 Mycosis fungoides, unspecified site, extranodal and solid organ sites;
202.18 Mycosis fungoides involving lymph nodes of multiple sites;
202.7 Peripheral T cell lymphoma, unspecified site, extranodal and solid organ sites

ICD10:
C83.30 Diffuse large B-cell lymphoma, unspecified site;
C83.38 Diffuse large B-cell lymphoma, lymph nodes of multiple sites;
C83.52 Lymphoblastic (diffuse) lymphoma, intrathoracic lymph nodes;
C83.79 Burkitt lymphoma, extranodal and solid organ sites;
C85.90 Non-Hodgkin lymphoma, unspecified, unspecified site;

Other Lymphoma
ICD 9:
202.8 Other malignant lymphomas, unspecified site, extranodal and solid organ sites;
202.82 Other malignant lymphomas involving intrathoracic lymph nodes

Myelodysplastic Syndrome/Myeloproliferative Neoplasm:
ICD9:
238.71 Essential thrombocythemia
238.72 Low grade myelodysplastic syndrome lesions
238.73 High grade myelodysplastic syndrome lesions
238.75 Myelodysplastic syndrome, unspecified
289.83 Myelofibrosis;

ICD10:
C93.30 Juvenile myelomonocytic leukemia, not having achieved remission;
C93.31 Juvenile myelomonocytic leukemia, in remission;
C94.6 Myelodysplastic disease, not classified;
D46.9 Myelodysplastic syndrome, unspecified;
D46.C Myelodysplastic syndrome with isolated del(5q) chromosomal abnormality;
D46.Z Other myelodysplastic syndromes;
D47.1 Chronic myeloproliferative disease

Solid Tumor:
ICD 9:
158 Malignant neoplasm of retroperitoneum;
162.9 Malignant neoplasm of bronchus and lung, unspecified;
164.3 Malignant neoplasm of posterior mediastinum;
164.9 Malignant neoplasm of mediastinum, part unspecified;
170.2 Malignant neoplasm of vertebral column, excluding sacrum and coccyx;
170.6 Malignant neoplasm of pelvic bones, sacrum, and coccyx;
170.7 Malignant neoplasm of long bones of lower limb;
171 Malignant neoplasm of connective and other soft tissue of head, face, and neck;
171.5 Malignant neoplasm of connective and other soft tissue of abdomen;
186.9 Malignant neoplasm of other and unspecified testis;
189 Malignant neoplasm of kidney, except pelvis;
190.5 Malignant neoplasm of retina;
191.5 Malignant neoplasm of ventricles;
191.6 Malignant neoplasm of cerebellum NOS;
191.7 Malignant neoplasm of brain stem;
191.8 Malignant neoplasm of other parts of brain;
191.9 Malignant neoplasm of brain, unspecified;
192.2 Malignant neoplasm of spinal cord;
192.3 Malignant neoplasm of spinal meninges;
192.9 Malignant neoplasm of nervous system, part unspecified;
194 Malignant neoplasm of adrenal gland;
194.4 Malignant neoplasm of pineal gland;
195 Malignant neoplasm of head, face, and neck;
195.2 Malignant neoplasm of abdomen;
209.2 Malignant carcinoid tumor of unknown primary site;
237.5 Neoplasm of uncertain behavior of brain and spinal cord;

239.2 Neoplasm of unspecified nature of bone, soft tissue, and skin;
239.6 Neoplasm of unspecified nature of brain;
239.7 Neoplasm of unspecified nature of endocrine glands and other parts of nervous system

ICD10:
C48.0 Malignant neoplasm of retroperitoneum;
C62.91 Malignant neoplasm of right testis, unspecified whether descended or undescended;
C71.0 Malignant neoplasm of cerebrum, except lobes and ventricles;
C71.3 Malignant neoplasm of parietal lobe;
C71.6 Malignant neoplasm of cerebellum;
C71.9 Malignant neoplasm of brain, unspecified;
C74.90 Malignant neoplasm of unspecified part of unspecified adrenal gland;
C74.91 Malignant neoplasm of unspecified part of right adrenal gland;
C74.92 Malignant neoplasm of unspecified part of left adrenal gland;
C75.3 Malignant neoplasm of pineal gland;
C76.1 Malignant neoplasm of thorax;
C76.2 Malignant neoplasm of abdomen;
D48.0 Neoplasm of uncertain behavior of bone and articular cartilage;
D49.6 Neoplasm of unspecified behavior of brain

Non-Malignant Hematologic Disease:

Sickle-cell anemia, Thalassemia, and other inherited anemia disorders
ICD9:
282.3 Other hemolytic anemias due to enzyme deficiency;
282.44 Beta Thalassemia;
282.6 Sickle-cell disease, unspecified;
282.61 Hb-SS disease without crisis;
282.62 Hb-SS disease with crisis;
282.68 Other sickle-cell disease without crisis;

ICD10:
D55.8 Other anemias due to enzyme disorders
D56.1 Beta thalassemia;
D56.9 Thalassemia, unspecified;
D57.1 Sickle-cell disease without crisis

Aplastic anemia and bone marrow failure syndromes
ICD9:
284.01 Constitutional red blood cell aplasia;
284.09 Other constitutional aplastic anemia;
284.89 Other specified aplastic anemias;
284.9 Aplastic anemia, unspecified;
285.8 Other specified anemias

ICD10:

D61.09Other constitutional aplastic anemia;
D61.3 Idiopathic aplastic anemia;
D61.89 Other specified aplastic anemias and other bone marrow failure syndromes;
D61.9 Aplastic anemia, unspecified

Immunodeficiency Disorders:
<u>ICD9:</u>
279.1 Immunodeficiency with predominant T-cell defect, unspecified;
279.12 Wiskott-Aldrich Syndrome;
279.2 Combined Immunity Deficiency;
288.01 Congenital neutropenia;
288.1 Functional disorders of polymorphonuclear neutrophils;
288.2 Genetic anomalies of leukocytes

<u>ICD10:</u>
D83.9 Common variable immunodeficiency, unspecified

Non-Malignant Other Disease:
<u>ICD9:</u>
277.5 Mucopolysaccharidosis;
288.4 Hemophagocytic syndromes;
701 Circumscribed scleroderma;
710.1 Systemic sclerosis;
756.52 Osteopetrosis
